# Unveiling the genomic landscape of *Salmonella enterica* serotypes Typhimurium, Newport, and Infantis in Latin American surface waters: a comparative analysis

**DOI:** 10.1128/spectrum.00047-24

**Published:** 2024-03-28

**Authors:** Zhao Chen, Magaly Toro, Andrea I. Moreno-Switt, Aiko D. Adell, Enrique J. Delgado-Suárez, Raquel R. Bonelli, Celso J. B. Oliveira, Angélica Reyes-Jara, Xinyang Huang, Brett Albee, Christopher J. Grim, Marc Allard, Sandra M. Tallent, Eric W. Brown, Rebecca L. Bell, Jianghong Meng

**Affiliations:** 1 Joint Institute for Food Safety and Applied Nutrition and Center for Food Safety and Security Systems, University of Maryland, College Park, Maryland, USA; 2 Instituto de Nutrición y Tecnología de los Alimentos, Universidad de Chile, Santiago, Chile; 3 Escuela de Medicina Veterinaria, Facultad de Ciencias Biológicas, Pontificia Universidad Católica de Chile, Santiago, Chile; 4 Escuela de Medicina Veterinaria, Facultad de Ciencias de la Vida, Facultad de Agronomía y Sistemas Naturales, Facultad de Ciencias Biológicas y Facultad de Medicina, Universidad Andrés Bello, Santiago, Chile; 5 Facultad de Medicina Veterinaria y Zootecnia, Universidad de Nacional Autónoma de México, Mexico City, Mexico; 6 Instituto de Microbiologia Paulo de Góes, Universidade Federal do Rio de Janeiro, Rio de Janeiro, Brazil; 7 Departamento de Zootecnia, Universidade Federal da Paraíba, Areia, Brazil; 8 Department of Nutrition and Food Science, University of Maryland, College Park, Maryland, USA; 9 Center for Food Safety and Applied Nutrition, United States Food and Drug Administration, College Park, Maryland, USA; Agriculture and Agri-Food Canada, Lacombe, Canada

**Keywords:** surface water, *Salmonella enterica*, whole-genome sequencing, multidrug resistance, integron, biomarker, genetic relatedness

## Abstract

Surface waters are considered ecological habitats where *Salmonella enterica* can persist and disseminate to fresh produce production systems. This study aimed to explore the genomic profiles of *S. enterica* serotypes Typhimurium, Newport, and Infantis from surface waters in Chile, Mexico, and Brazil collected between 2019 and 2022. We analyzed the whole genomes of 106 *S*. Typhimurium, 161 *S*. Newport, and 113 *S*. Infantis isolates. Our phylogenetic analysis exhibited distinct groupings of isolates by their respective countries except for a notable case involving a Chilean *S*. Newport isolate closely related to two Mexican isolates, showing 4 and 13 single nucleotide polymorphisms of difference, respectively. The patterns of the most frequently detected antimicrobial resistance genes varied across countries and serotypes. A strong correlation existed between integron carriage and genotypic multidrug resistance (MDR) across serotypes in Chile and Mexico (*R* > 0.90, *P* < 0.01), while integron(s) were not detected in any of the Brazilian isolates. By contrast, we did not identify any strong correlation between plasmid carriage and genotypic MDR across diverse countries and serotypes.

**IMPORTANCE:**

Unveiling the genomic landscape of *S. enterica* in Latin American surface waters is pivotal for ensuring public health. This investigation sheds light on the intricate genomic diversity of *S. enterica* in surface waters across Chile, Mexico, and Brazil. Our research also addresses critical knowledge gaps, pioneering a comprehensive understanding of surface waters as a reservoir for multidrug-resistant *S. enterica*. By integrating our understanding of integron carriage as biomarkers into broader MDR control strategies, we can also work toward targeted interventions that mitigate the emergence and dissemination of MDR in *S. enterica* in surface waters. Given its potential implications for food safety, this study emphasizes the critical need for informed policies and collaborative initiatives to address the risks associated with *S. enterica* in surface waters.

## INTRODUCTION

*Salmonella*, an enteric pathogen with over 2,600 serotypes, has long been associated with foodborne illnesses and public health challenges (1). Among these, *Salmonella enterica* subspecies *enterica* encompasses more than 1,500 serotypes, which is responsible for more than 99% of human salmonellosis (2). Historically, the primary concern surrounding *S. enterica* infections has revolved around contaminated food products (3). However, our understanding of the ecological adaptability of *S. enterica* has broadened in recent years to encompass surface waters, which include a broad spectrum of water bodies such as rivers, lakes, ponds, and irrigation canals (4–6). These aquatic ecosystems have emerged as significant ecological niches where *S. enterica* can survive and potentially contribute to its ongoing presence in fresh produce production systems (7).

Remarkably, *S. enterica* can persist in diverse aquatic ecosystems, adapting to varying temperatures, nutrient levels, and the presence of competing microorganisms (8). This adaptability allows *S. enterica* to maintain a reservoir in surface waters. The recognition of the presence of *S. enterica* in surface waters has given rise to emerging public health issues. Surface waters are not only habitats for *S. enterica* but also are interconnected with agricultural production systems (5, 9). The presence of *S. enterica* in surface waters is a matter of concern due to its potential implications for food safety, particularly in the context of fresh produce. As surface waters are commonly used for irrigation and may come into contact with fresh produce, the risk of contamination with *S. enterica* increases (10).

*S. enterica* strains found in surface waters may possess distinctive genomic features, possibly altering their antimicrobial resistance (AMR) and pathogenicity (11). In Latin America, a region characterized by rich biodiversity and varying environmental conditions, the presence of *S. enterica* in surface waters has become a subject of heightened interest. Prior research has unveiled the high prevalence and genetic variability of *S. enterica* in surface waters in Latin America, including Chile, Mexico, and Brazil (12–17). These three countries are not only major food producers but also leading exporters of fresh produce in the region (18). The reliance on surface waters for agricultural practices creates a critical interface between *S. enterica*, the environment, and the food supply chain. *S. enterica* originating from contaminated surface waters in these countries can potentially contaminate fresh produce, affecting not only local populations but also posing risks to international trade and worldwide public health. It is, therefore, imperative to extend our knowledge of *S. enterica* circulating in these waters, as they have been less studied compared to traditional food sources. The study of *S. enterica* in surface waters becomes pivotal, considering that these waters serve as a reservoir for the pathogen and may contribute to its dissemination through the food web.

In an era marked by advances in genomics and molecular epidemiology, whole-genome sequencing (WGS) has emerged as a powerful tool for unraveling the genomic intricacies of human pathogens (19). Our study, therefore, employed WGS to shed light on the genomic diversity of *S*. Typhimurium, Newport, and Infantis from surface waters in Chile, Mexico, and Brazil. These serotypes were among the top 10 most prevalent serotypes for each country (our unpublished data). When considering isolates from three countries collectively, they emerged as the top three most prevalent serotypes. Moreover, by comparing the genomic attributes of *S. enterica* in surface waters from distinct Latin American regions, this research also delved into the genetic relationships among isolates of each serotype. In addition, it can contribute to future strategies for mitigating the risks associated with *S. enterica* in surface waters, ultimately aiding in the development of more effective surveillance and intervention measures.

## MATERIALS AND METHODS

### *S*. Typhimurium, Newport, and Infantis isolates

*S*. Typhimurium (*n* = 349), Newport (*n* = 339), and Infantis (*n* = 301) isolates were collected from various surface water sources such as rivers, dams, lakes, ponds, and irrigation canals in Chile, Mexico, and Brazil from 2019 to 2022. The sampling strategies and the characteristics of the sampling sites were described by Toro et al. (17) and Ballesteros-Nova et al. (15). The isolates were collected as part of a collaborative surveillance initiative involving the University of Maryland, partner universities in Chile, Mexico, and Brazil, and the United States Food and Drug Administration (FDA). To avoid biases in subsequent genomic analyses, clonal *S*. Typhimurium, Newport, and Infantis isolates were identified and excluded from this study. Clonal isolates were defined as those derived from the same sample and grouped in the same single nucleotide polymorphism (SNP) cluster according to the National Center for Biotechnology Information (NCBI) Pathogen Detection database (15). In addition, in alignment with the criteria set by the FDA, these isolates were mandated to have an SNP distance of ≤20 (20). This cut-off was established based on a thorough examination of the published literature, particularly focusing on the maximum pairwise SNPs observed in investigations related to foodborne outbreaks and contamination events. After excluding clonal isolates, we included 106 *S*. Typhimurium, 161 *S*. Newport, and 112 *S*. Infantis isolates from surface waters in Chile, Mexico, and Brazil in the present study (Table 1; Table S1).

**TABLE 1:** Numbers of *Salmonella enterica* serotypes Typhimurium, Newport, and Infantis isolates from Latin American surface waters

Serotype	Number of isolates			
	Chile	Mexico	Brazil	Combined
Typhimurium	62	28	16	106
Newport	51	73	37	161
Infantis	72	22	18	112

### DNA extraction

*S*. Typhimurium, Newport, and Infantis isolates were streaked onto trypticase soy agar (TSA; Fisher Scientific Inc., Hampton, NH). After a 24-h incubation at 35°C, one single colony was transferred into tryptic soy broth (TSB; Fisher Scientific Inc.). The TSB culture was then allowed to grow overnight at 35°C. Genomic DNA was extracted using the Maxwell RSC cultured cells DNA kit (Promega Corporation, Madison, WI) on the Maxwell RSC 48 instrument (Promega Corporation). Once extracted, the genomic DNA samples were stored at 4°C until they were ready for use. The concentration of DNA in each sample was measured using the Qubit 1× dsDNA broad range assay kit (Fisher Scientific Inc.) on the Qubit 3.0 fluorometer (Fisher Scientific Inc.).

### Library preparation and WGS

Libraries were prepared on the Sciclone G3 NGSx iQ workstation (PerkinElmer, Inc., Waltham, MA) using the Illumina DNA prep kit in conjunction with IDT for Illumina DNA/RNA UD indexes (Illumina Inc., SanDiego, CA). WGS was performed on the NextSeq 2000 platform (Illumina Inc.) with the NextSeq 1000/2000 P2 reagents (300 Cycles) (Illumina Inc.) with 2 × 150 bp paired-end chemistry.

### Data pre-processing and genome assembly

Raw reads were subjected to trimming using Trimmomatic 0.39 (21). Specifically, we employed the SLIDINGWINDOW operation with a window size of four bases for averaging and a minimum average quality score of 20. Subsequently, the trimmed reads were utilized for genome assembly, which was performed using SPAdes 3.15.5 (22), following the default settings. This assembly process involved the use of *k*-mers at sizes 21, 33, and 55, along with careful correction to improve accuracy. To examine the quality of each assembly, a thorough quality check was carried out using QUAST 5.2.0 (23). Contigs with short lengths (<1,000 bp) and/or low coverages (<30×) were excluded from each assembly to minimize the inclusion of potential contaminants.

### Identifications of AMR determinants, plasmids, integrons, and virulence genes

AMRFinderPlus 3.11.14 was used to detect AMR determinants [AMR genes (ARGs) and point mutations] (24), with a minimum identity threshold of −1 and a minimum coverage of 50%. To streamline the terminology, we defined “genotypically antimicrobial-resistant” isolates with at least one AMR determinant as “resistant.” Similarly, for “genotypically multidrug-resistant” isolates, we defined them as “multidrug-resistant” when they exhibited AMR determinants associated with at least three distinct antimicrobial classes. Plasmids were identified using Staramr 0.9.1 (25), which compared the sequences to known plasmid sequences integrated with the PlasmidFinder database (26). This process utilized a minimum identity of 98% and a minimum coverage of 60%. Integron identification was carried out using IntegronFinder 2.0.2 (27), with a clustering threshold of 4 kb. The analysis included a filter for the clusters of *attC* sites lacking integron integrases (CALINs) with a specified threshold, and the *attC* size was constrained to a maximum of 200 bp and a minimum of 40 bp for accurate detection. We conducted the Pearson correlation analysis using SigmaPlot 15 (Systat Software Inc., San Jose, CA) to assess (i) the correlation between the presence/absence of plasmid(s) or integron(s) and genotypic AMR or multidrug resistance (MDR) for each isolate and (ii) the correlation between the proportion of plasmid- or integron-carrying isolates and the proportion of resistant or multidrug-resistant isolates among all isolates. The correlation matrix was plotted using the “ggplot2” 3.4.4 (28), “corrplot” 0.92 (29), “ggplotify” 0.1.2 (30), and “ggcorrplot” 0.1.4.1 (31) R packages (R 4.3.2). Linear regression was conducted and visualized with the “ggplot2” and “ggrepel” 0.9.4 R packages (32) when a strong correlation existed [correlation coefficient (*R*) ≥ 0.80 or ≤−0.80, *P* < 0.05] (33). For the detection of virulence genes, we employed ABRicate 1.0.0, utilizing known gene sequences from the Virulence Factors Database (VFDB) (34). The criteria for this detection included a minimum identity of 80% and a minimum coverage of 60%.

### Multilocus sequence typing

We conducted multilocus sequence typing (MLST) using mlst 2.23.0 (35; https://github.com/tseemann/mlst). This tool integrates components from the PubMLST database and performs scans on whole genomes against traditional PubMLST typing schemes that rely on seven housekeeping genes. Specific criteria were applied for the analysis, including a minimum identity threshold for the full allele of 95%, a minimum coverage requirement for the partial allele of 10%, and a minimum score to match a scheme of 50. As a result, mlst also reported the sequence types (STs) obtained from the analysis.

### Whole-genome phylogeny

SNPs were called and filtered for each serotype using the CFSAN SNP pipeline (36). *S*. Typhimurium LT2 (RefSeq assembly accession: GCF_000006945.2), Newport CDC 2010K-2159 (RefSeq assembly accession: GCF_000973685.2), and Infantis FSIS1502916 (RefSeq assembly accession: GCF_001931575.1) served as the reference genomes for the whole-genome phylogenetic analysis of *S*. Typhimurium, Newport, and Infantis isolates, respectively. Following SNP calling, we employed FastTree 2.1.11 (37) to construct whole-genome maximum-likelihood phylogenetic trees based on the generalized time-reversible model. Each inferred whole-genome phylogeny was then visualized as a rooted rectangular phylogram using iTOL 6.7.4 (38).

### Core-genome MLST

The core-genome MLST (cgMLST) analysis was conducted using cgMLSTFinder 1.2 (39; https://cge.cbs.dtu.dk/services/cgMLSTFinder/). This analysis utilized the core-genome database for *Salmonella* retrieved from EnteroBase (40), which encompasses 3,002 loci. Subsequently, a minimum spanning tree, based on the allelic profiles for each serotype, was constructed using GrapeTree 1.5.0 (41).

### Pan-genome

Whole genomes were annotated using Prokka 1.14.5 (42), with a locus tag counter increment of one, a minimum contig size of 200, and a similarity e-value cut-off of 0.000001. Afterward, we conducted pan-genome analysis utilizing Roary 3.13.0 (43), with a minimum percentage identity for blastp of 95% and a maximum limit of 50,000 clusters. We utilized the annotated genomes as input for Roary, enabling us to determine the quantities of core and accessory (soft-core, shell, and cloud) genes: core genes are present in 99% ≤ n ≤ 100%; soft-core genes are present in 95% ≤ n < 99%; shell genes are present in 15% ≤ n < 95%; cloud genes are present in 0% ≤ n < 15%. Heatmaps displaying the counts of core and accessory genes were generated with the “pheatmap” 1.0.12 R package (44). Area-proportional Venn diagrams illustrating the core and accessory genes were created using the “VennDiagram” 1.7.3 R package (45). Parsnp 1.7.4 was used to execute core-genome SNP alignment (46). This procedure involved the automated recruitment of the reference sequence and required the inclusion of all genomes for the analysis. The pan-genome results were visualized employing the Roary plots module to construct a matrix showcasing the presence/absence of core and accessory genes in the context of the core-genome phylogenetic tree. In addition, a pan-genome pie chart was generated to provide insights into the composition of core, soft-core, shell, and cloud genes for each serotype. The t-test was conducted using SigmaPlot 15 to determine if significant differences (*P* < 0.05) existed among countries.

## RESULTS AND DISCUSSION

### AMR determinants

**Fig 1:**
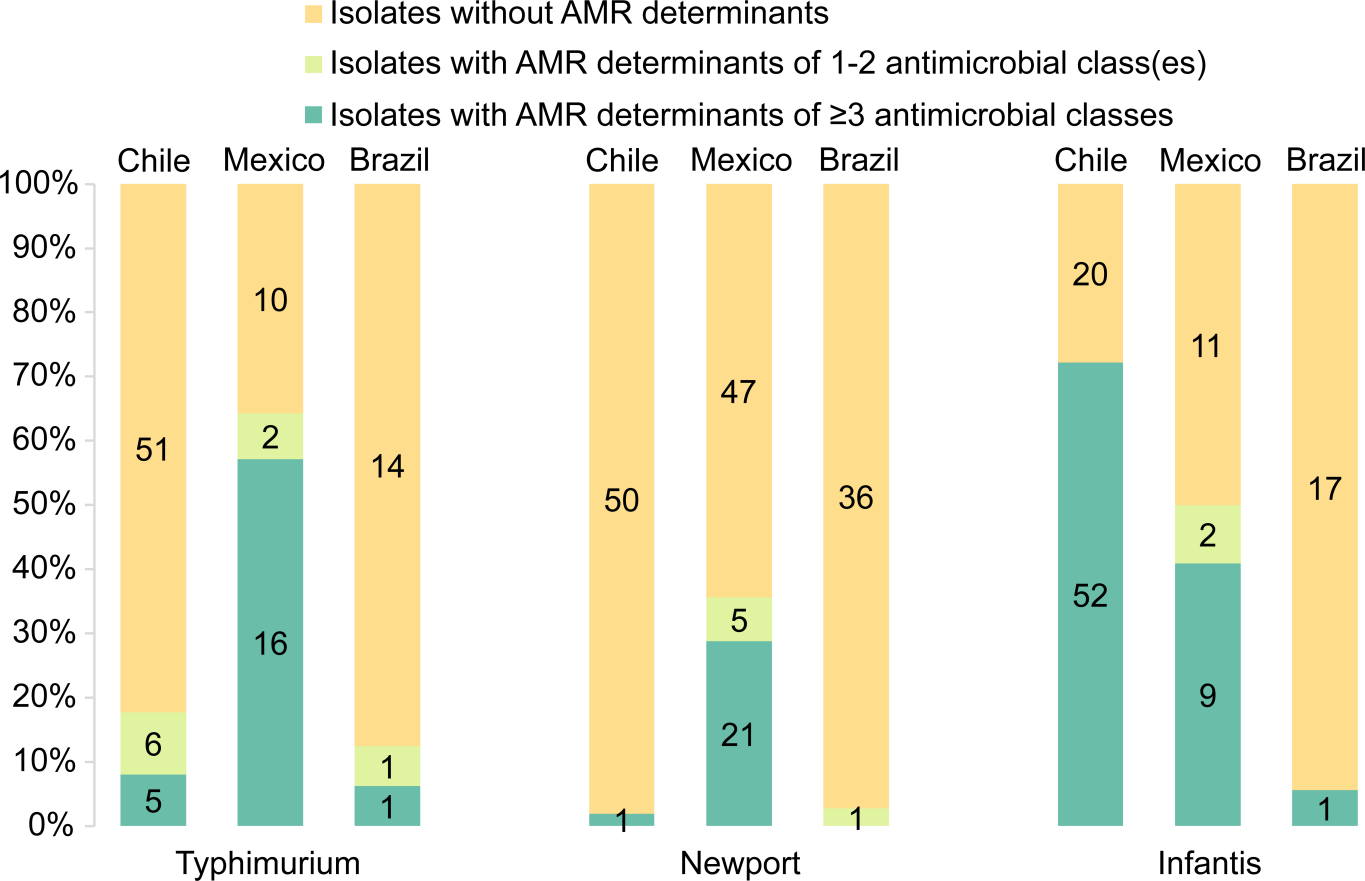
Genotypic antimicrobial resistance (AMR) of *Salmonella enterica* serotypes Typhimurium, Newport, and Infantis isolates from Latin American surface waters. The number noted on each category indicates the number of isolates in the category.

The presence of ARGs and point mutation among *S*. Typhimurium isolates from Chile, Mexico, and Brazil was observed in 17.7 (11/62), 64.3 (18/28), and 12.5% (2/16) of cases, respectively (Fig. 1). For *S*. Newport isolates from these countries, the presence of ARGs and point mutation was 2.0 (1/51), 35.6 (26/73), and 2.7% (1/37), respectively. In the case of *S*. Infantis isolates, 72.6 (52/72), 50.0 (11/22), and 5.6% (1/18) from Chile, Mexico, and Brazil contained these features, respectively. The predominant ARGs exhibited variations among countries and serotypes (Table 2).

**TABLE 2:** Predominant antimicrobial resistance genes among genotypically antimicrobial-resistant *Salmonella enterica* serotypes Typhimurium, Newport, and Infantis isolates from Latin American surface waters

Serotype	Predominant antimicrobial resistance genes			
	Chile	Mexico	Brazil	Combined
Typhimurium	*sul2* (5/11)	*floR* (15/18)	*aph(3'')-Ib*, *aph(6)-Id*, *sul2*, *tet(A*), *qnrB19* (1/2)	*aph(3'')-Ib*, *aph(6)-Id*, *sul2*, *tet(A)* (19/31)
Newport	*aadA2*, *blaCARB-2*, *dfrA1*, *floR*, *mph(A*), *qacE*, *qnrA1*, *sul1*, *tet(A)* (1/1)	*dfrA1*, *floR*, *mph(A*), *qacEdelta1*, *sul1*, *tet(A)* (21/26)	*qnrB19* (1/1)	*dfrA1*, *floR*, *mph(A*), *qacEdelta1*, *sul1*, *tet(A)* (22/28)
Infantis	*tet(A)* (53/53)	*aadA1*, *qacEdelta1*, *tet(A)* (9/11)	*blaTEM-1*, *floR*, *tet(A)* (1/1)	*tet(A)* (63/65)

^*a*^ Data in parentheses indicate the number of isolate(s) harboring the antimicrobial resistance gene(s)/the number of genotypically antimicrobial-resistant isolate(s).

A point mutation in *gyrA*_S83Y was the sole observed mutation in the quinolone resistance-determining region (QRDR) for *S*. Typhimurium and Infantis isolates from Chile and Mexico. None of the *S*. Newport isolates, regardless of the country of origin, exhibited the mutation (Chile: 0/1; Mexico: 0/26; Brazil: 0/1), although *gyrA* mutations in *S*. Newport have previously been reported (47–49). The mutation was present in 3.2 (2/62) and 10.7% (3/28) of *S*. Typhimurium isolates from Chile and Mexico, respectively. For *S*. Infantis isolates from Chile and Mexico, the mutation was detected in 72.2 (52/72) and 27.3% (6/22) of cases, respectively. By contrast, none of the *S*. Typhimurium (0/2) and Infantis (0/1) isolates from Brazil carried the mutation. Meanwhile, 9.1 (1/11) and 16.7% (3/18) of resistant *S*. Typhimurium isolates from Chile and Mexico exhibited the mutation. By contrast, among resistant *S*. Infantis isolates, the mutation was present in all Chilean isolates and 54.5% (6/11) of Mexican isolates.

Among resistant isolates, *S*. Typhimurium isolates exhibited MDR proportions of 45.5% (5/11) in Chile, 88.9% (16/18) in Mexico, and 50.0% (1/2) in Brazil (Fig. 1). For *S*. Newport isolates, these rates were 100.0% (1/1) in Chile, 80.8% (21/26) in Mexico, and 0.0% (0/1) in Brazil. In the case of *S*. Infantis isolates, the prevalence of MDR was 100.0% (52/52) in Chile, 81.8% (9/11) in Mexico, and 100.0% (1/1) in Brazil, signifying a high level of MDR among these isolates. A point mutation in *gyrA*_S83Y was present in 40.0 (2/5) and 18.8% (3/16) of multidrug-resistant *S*. Typhimurium isolates from Chile and Mexico, respectively. By contrast, multidrug-resistant *S*. Infantis isolates were observed to have a higher mutation prevalence, with 100.0 (52/52) and 66.7% (6/9) for Chile and Mexico, respectively. The mutation was not detected in multidrug-resistant *S*. Newport isolates (Chile: 0/1; Mexico: 0/21). None of the multidrug-resistant Brazilian isolates with genotypic AMR had the mutation (*S*. Typhimurium: 0/1; *S*. Infantis: 0/1). Our results reveal that MDR, an important concern for public health, is notably high among resistant *S*. Infantis isolates from all countries. While *S*. Typhimurium isolates from Mexico had a high proportion of MDR, *S*. Newport isolates from Brazil displayed the lowest MDR rates.

The substantial occurrence of *tet(A*) in *S. enterica* isolates from Chile, Mexico, and Brazil aligns with earlier reports that underscore the widespread prevalence of tetracycline resistance in bacterial populations, including *S. enterica*, within surface waters in these regions (50–53). However, our results also demonstrate that the prevalence of AMR determinants varied significantly among both serotypes and within isolates of the same serotype across Chile, Mexico, and Brazil, highlighting the complex interaction of serotype-, strain-, and country-specific AMR profiles among these isolates. Chile has been identified as one of the top five countries contributing significantly to global antimicrobial consumption in animal production in 2020 (54). In addition, projections indicate that by 2030, Mexico and Brazil are expected to join the top five countries with the largest shares of global antimicrobial consumption in animal production (55).

Noticeably, antimicrobial usage (AMU) in agricultural practices, especially animal husbandry, can directly impact the prevalence of AMR (56–59). Resistant strains may emerge in response to the selective pressure imposed by routine AMU in veterinary medicine. Hence, distinct AMR patterns among isolates of the same serotype from Chile, Mexico, and Brazil potentially reflect the variation in local practices in AMU, which emphasizes the importance of considering geographical origin when evaluating the risk of resistant *S. enterica*. For instance, Mexican isolates exhibited a high prevalence of ARGs linked to phenicol, trimethoprim, and sulfonamide resistance. The approval of trimethoprim-sulfamethoxazole as a broad-spectrum antimicrobial for treating bacterial infections in livestock in Mexico aligns with our frequent detection of corresponding ARGs, including *dfrA1* and *sul1* (60). Moreover, despite the discontinuation of chloramphenicol, other phenicol-based antimicrobials, such as florfenicol, remain registered and utilized in Mexico.

Nevertheless, it is crucial to exercise caution when attempting to correlate AMU with observed AMR patterns, primarily due to the significant challenge posed by the limited availability of comprehensive and specific data on local AMU within the sampled regions. Obtaining accurate information about the types, quantities, and frequencies of antimicrobials used in animal husbandry in specific regions within Chile, Mexico, and Brazil is often challenging. The multifaceted nature of AMR involves intricate interactions between environmental, genetic, and anthropogenic factors, making it even harder to pinpoint the direct impact of AMU on AMR patterns without detailed and consistent usage data. There is, thus, an urgent need for enhanced monitoring systems to track AMU in animal husbandry in these countries, which is crucial for assessing its impact on public health and developing targeted interventions.

Notably, the AMR features also differed among serotypes and within isolates of the same serotype in each country. Each *S. enterica* serotype may have distinct genetic characteristics, including the presence of specific AMR determinants, leading to variations in AMR profiles (61). The genetic diversity observed in isolates of the same serotype may be attributed, in part, to the wide geographical distribution of the sampled areas within each country. By collecting samples from various regions, including both urban and rural environments, we aimed to capture the diverse ecological niches where *S. enterica* may persist. The inclusion of samples from different locales increases the likelihood of encountering distinct bacterial populations, contributing to the observed genetic diversity. The variations in AMR features within isolates of the same serotype in each country could, thus, be due to local selection pressures. Factors such as differences in AMU practices, agricultural practices, and environmental conditions may create unique selective environments favoring the emergence of specific AMR mechanisms.

### Plasmid(s)

Notably, a significant proportion of *S*. Typhimurium isolates in all three countries were found to carry plasmids (Chile: 96.8%, 60/62; Mexico: 89.3%, 25/28; Brazil: 100.0%, 16/16), with the highest prevalence observed in Brazil (Table S2). Conversely, plasmid occurrence in *S*. Newport isolates was notably lower (Chile: 7.8%, 4/51; Mexico: 41.1%, 30/73; Brazil: 2.7%, 1/37), with the least frequency in Brazilian isolates (Table S2). However, the prevalence of plasmids in *S*. Infantis isolates exhibited variation (Chile: 76.4%, 55/72; Mexico: 36.4%, 8/22; Brazil: 5.6%, 1/18) (Table S2). These findings suggest that the carriage of plasmids varied not only among serotypes but was also influenced by geographical factors. The high prevalence of plasmids in *S*. Typhimurium isolates, especially in Chile and Brazil, is a noteworthy observation.

It is important to highlight that all resistant *S*. Typhimurium (Chile: 11/11; Mexico: 18/18; Brazil: 2/2) and Newport (Chile: 1/1; Mexico: 26/26; Brazil: 1/1) isolates from the three countries carried plasmid(s). All resistant *S*. Infantis isolates from Chile carried plasmid(s) (52/52), while 72.7% (8/11) of the resistant *S*. Infantis isolates from Mexico exhibited plasmid presence. However, plasmids were not present in the sole resistant *S*. Infantis isolate from Brazil. Our Pearson correlation analysis did not reveal any strong correlation between plasmid carriage and genotypic AMR across various countries and serotypes (Fig. S1A and B). Isolates carrying plasmid(s) that bear ARGs are more likely to exhibit resistance to antimicrobials targeted by those genes (62). In instances where isolates carry plasmid(s) devoid of ARGs specific to certain antimicrobials, they may remain susceptible to those antimicrobials. A comprehensive study examining 150,767 *S*. *enterica* genomes across 1,204 distinct serotypes revealed that most plasmids in *S. enterica* are not involved in the dissemination of ARGs (63).

All multidrug-resistant *S*. Typhimurium (Chile: 5/5; Mexico: 16/16; Brazil: 1/1) and Newport (Chile: 1/1; Mexico: 21/21) isolates from the three countries were found to contain plasmid(s). All *S*. Infantis isolates from Chile (52/52) and 66.7% (6/9) of *S*. Infantis isolates from Mexico with MDR were found to harbor plasmid(s). However, plasmids were absent in the only multidrug-resistant *S*. Infantis isolates from Brazil. Our Pearson correlation analysis did not identify any strong correlation between plasmid carriage and genotypic MDR across diverse countries and serotypes. Our study relied on Illumina short reads, and the draft-genome nature of the data hindered the ability to accurately pinpoint the specific genomic locations of ARGs (64). Further investigations should necessitate long-read sequencing techniques to obtain complete genomes, which would enable in-depth exploration of the structural attributes and locations (chromosome- or plasmid-borne) of ARGs.

### Integron(s)

Integron(s) were detected in 3.2% (2/62) and 57.1% (16/28) of *S*. Typhimurium isolates from Chile and Mexico, respectively (Table S3). For *S*. Newport isolates, integron(s) were found in 2.0% (1/51) and 17.8% (13/73) from Chile and Mexico, respectively (Table S3). In the case of *S*. Infantis isolates, integron(s) were identified in 72.2% (52/72) and 40.9% (9/22) from Chile and Mexico, respectively (Table S3). None of the Brazilian isolates harbored integron(s), regardless of serotype.

**Fig 2:**
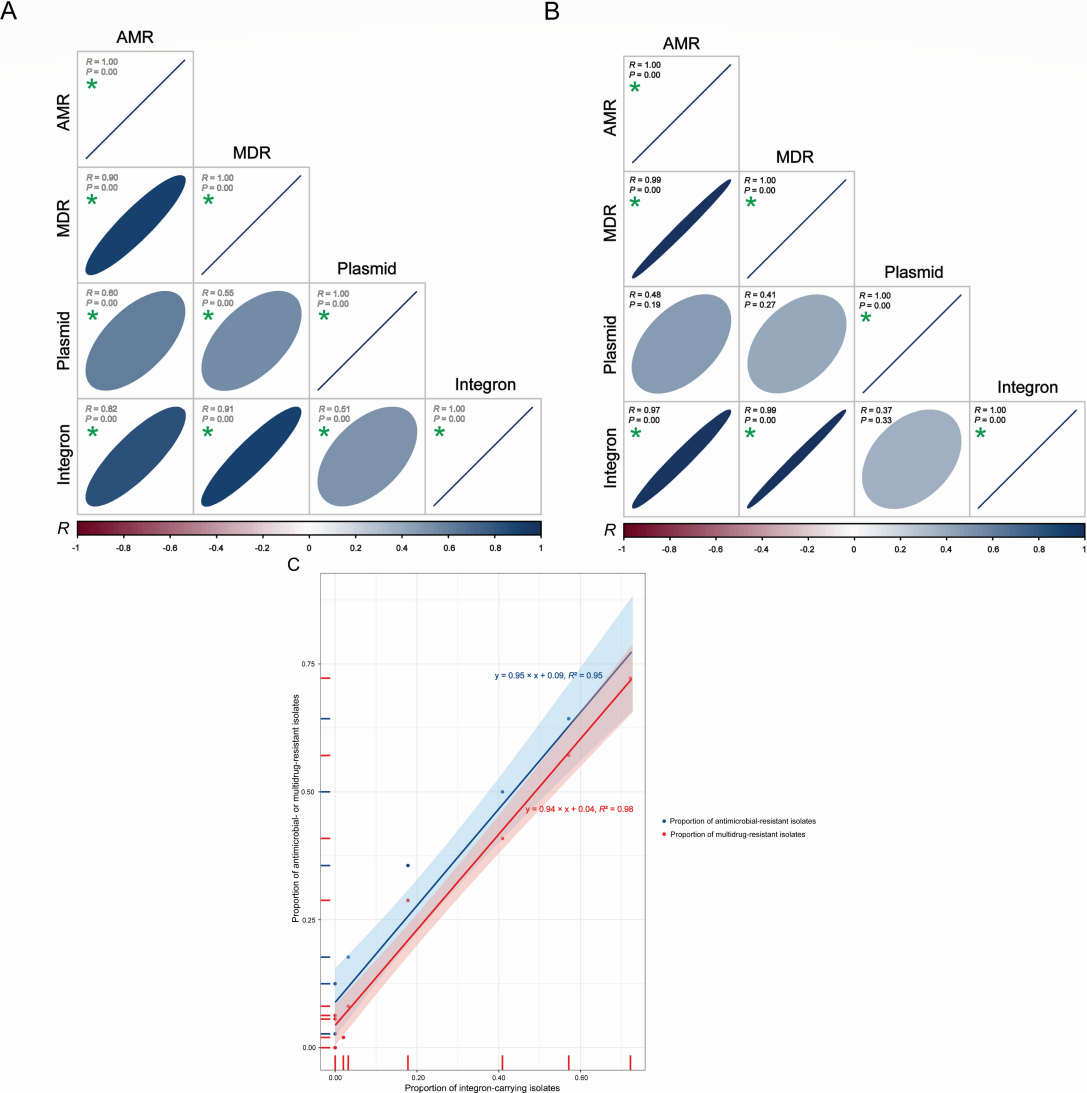
Correlations between integron carriage and genotypic antimicrobial resistance (AMR) or multidrug resistance (MDR) of *Salmonella enterica* serotypes Typhimurium, Newport, and Infantis isolates from Latin American surface waters: (A) Pearson correlation between the presence/absence of plasmid(s) or integron(s) and genotypic AMR or MDR for each isolate; (B) Pearson correlation between the proportion of isolates carrying plasmid(s) or integron(s) and the proportion of those with genotypic AMR or MDR among all isolates. The correlation coefficient (R) is presented in an ellipse by a coloring scheme from red (negative correlation) to blue (positive correlation). The size of each ellipse is negatively correlated with *R*. The combination labeled with a green asterisk shows a significant difference (*P* < 0.05)); (C) linear regression between the proportion of isolates carrying integron(s) and the proportion of those with genotypic AMR or MDR.

Integrons are genetic elements that can capture and express gene cassettes containing ARGs, which play a significant role in the dissemination of AMR among bacteria (65, 66). Most importantly, integron-mediated ARGs have previously been reported to contribute to the MDR of *S. enterica* (67, 68). It should be noted that all Chilean (*S*. Typhimurium: 2/2; *S*. Newport: 1/1; *S*. Infantis: 52/52) and Mexican (*S*. Typhimurium: 16/16; *S*. Newport: 13/13; *S*. Infantis: 9/9) isolates containing integron(s) were multidrug-resistant across serotypes (Fig. 1). Noticeably, our Pearson correlation analysis underscores a strong positive correlation between integron carriage and genotypic AMR, especially MDR, spanning diverse countries and serotypes (*R* > 0.80, *P* < 0.05) (Fig. 2A and B). We found a linear correlation between the proportion of *S*. Typhimurium, Newport, and Infantis isolates carrying integron(s) and the proportion of those with genotypic AMR (*R*² =0.95) (Fig. 2C). Interestingly, a more robust positive correlation existed between the proportion of *S*. Typhimurium, Newport, and Infantis isolates waters carrying integron(s) and the proportion of those with genotypic MDR across diverse countries and serotype (*R*² =0.98) (Fig. 2).

In this study, the observed strong correlation between integron carriage and genotypic MDR suggests a potential role of integrons in mediating MDR in *S. enterica*. The presence of integrons was consistently associated with genotypic MDR in *S*. Typhimurium, Newport, and Infantis isolates from diverse geographical regions. This correlation implies that the acquisition and maintenance of integrons can contribute significantly to the accumulation of ARGs in *S. enterica*, leading to a higher likelihood of MDR.

Recent studies have underscored the significance of integrons as predictive biomarkers for AMR in various settings. Barraud et al. (69) highlighted the potential of integrons as predictive markers for detecting AMR in acute sepsis, emphasizing their role in Gram-negative bacteria-positive blood cultures (70). Azizi et al. (71) evaluated integrons in *Acinetobacter baumannii* and identified class 1 integrons as biomarkers for MDR phenotypes in clinical situations. Similarly, the study by Hsiao et al. (72) associated class 1 integrons with ARG cassettes in *Pseudomonas aeruginosa*. Drawing parallels with these findings, our study emphasizes the crucial role of monitoring integron carriage as biomarkers to comprehend the potential for MDR development in *S. enterica* populations. This knowledge is pivotal for developing effective strategies to mitigate the spread of multidrug-resistant strains. Given the implications of integron carriage, our findings advocate for their incorporation into future surveillance initiatives. Specifically targeting integrons as biomarkers in surveillance strategies will enable the monitoring of integron-mediated MDR prevalence and evolution over time. In addition, optimizing water examination protocols becomes imperative to curtail the dissemination of integron-bearing *S. enterica*. Exploring specific gene cassettes within integrons and their correlations with MDR profiles can also provide valuable insights into the mechanisms driving MDR in *S. enterica*. The primary limitation in our study regarding the precise localization of ARGs and integrons stems from the use of Illumina short reads. Future research utilizing long-read sequencing techniques to obtain complete genomes has the potential to shed light on the structures and physical proximity of specific ARGs and integrons.

### Virulence genes

A total of 116 virulence genes were identified in *S*. Typhimurium isolates from all countries (Fig. S1A). Interestingly, we observed that some virulence genes were exclusively present in Chilean and Brazilian isolates. Specifically, *astA* encoding the heat-stable enterotoxin 1 was solely detected in one Brazilian isolate. Also, *cesT* encoding multi-effector chaperone was exclusively found in one Chilean isolate, and *gspI* encoding the general secretion pathway protein I was identified in just one Chilean isolate. Furthermore, it is noteworthy that *sspH1* encoding the type III secretion system effector SspH1 E3 ubiquitin ligase was carried by only two Chilean isolates and three Mexican isolates. In addition, *shdA* encoding the AIDA autotransporter-like protein was only identified in seven Chilean isolates, four Mexican isolates, and one Brazilian isolate. Noticeably, two isolates from Brazil and Mexico did not possess some virulence genes that were universally present in other isolates. Specifically, one Brazilian isolate lacked two genes consistently found in other isolates, including *sodCI* encoding the superoxide dismutase precursor (Cu-Zn) and *sseI/srfH* encoding the type III secretion system effector SseI/SrfH cysteine protease. In addition, one Mexican isolate did not contain *ssaI*, the gene encoding the type III secretion system inner rod protein SsaI.

In *S*. Newport isolates from all countries, up to 106 virulence genes were detected (Fig. S1B). It is worth noting the specific distribution of virulence genes among these isolates. For instance, *astA* was exclusively identified in 13 Chilean isolates. Also, *cdtB* encoding the cytolethal distending toxin B was found in only one isolate from Chile, and *cheY* encoding the chemotaxis protein CheY was observed in a sole isolate from Mexico. A subset of isolates from the three countries was devoid of genes consistently identified in other isolates. Specifically, *pipB* encoding the type III secretion system effector PipB was not detected in two Mexican isolates, and *ratB* encoding the putative outer membrane protein was notably absent in six Mexican and one Brazilian isolate. Furthermore, *sicP* encoding the chaperone for SptP and *sinH* encoding the intimin-like protein were not found in one Chilean isolate and three Mexican isolates, respectively. Lastly, *sspH2* encoding the type III secretion system effector SspH2 E3 ubiquitin ligase was lacking in five Mexican isolates.

Figure 1C illustrates that a combined total of 111 virulence genes were detected in *S*. Infantis isolates from all the countries. Notably, some isolates from Chile and Brazil lacked genes universally found in other isolates. Specifically, two Chilean isolates did not carry *ssek1* encoding the type III secretion system effector SseK1, while *ratB* was not detected in one isolate from Brazil.

The presence of genotypic MDR (aminoglycoside, beta-lactam, bleomycin, fosfomycin, lincosamide, phenicol, quaternary ammonium, quinolone, sulfonamide, tetracycline, and trimethoprim) and the absence of specific virulence genes in certain *S*. Typhimurium isolates, including six Chilean isolates and 17 Mexican isolates, indicate the potential fitness costs imposed by either ARGs or virulence genes (Fig. S1A). While the development of AMR may confer survival advantages in the presence of antimicrobials, it can lead to selective disadvantages in terms of bacterial virulence (73). The underlying mechanisms of such fitness costs are complex and multilayered (74). Although increased resistance to aminoglycoside, beta-lactam, and quinolone has been documented to be associated, either directly or indirectly, with attenuated virulence attributes of *S. enterica* (75–77), the current body of literature still lacks in-depth coverage of these biological compromises in *S. enterica*.

The missing virulence genes in these *S*. Typhimurium isolates encompass *gogB* (solely absent in Mexican isolates) responsible for encoding the type III secretion system effector GogB, *grvA* related to the Gifsy-2 prophage, the *pef* gene cluster encoding the plasmid-encoded fimbriae, *rck* involved in resistance to complement killing, and the *spv* gene cluster responsible for *Salmonella* plasmid virulence. Notably, several missing virulence genes are linked to mobile genetic elements (MGEs) such as prophages and plasmids. This process may be influenced by the movement of MGEs through horizontal gene transfer, where their loss or acquisition can result in changes in bacterial traits (78). The connection between missing virulence genes and their associated MGEs suggests a dynamic interplay in the genetic makeup of *S. enterica*, influenced by factors such as AMR, bacterial evolution, and the transfer of genetic materials. This dynamic nature of virulence genes and MGEs is a critical area for further research on bacterial adaptation and survival. To gain a comprehensive understanding of the fundamental mechanisms, additional virulence assessment would necessitate an in-depth exploration of whether the loss of these virulence genes could lead to actual virulence attenuation.

It is significant to mention that the specific fitness costs can vary depending on the strains and the types of antimicrobials encountered. The compromises were not always consistent since some resistant strains still maintained their virulence genes, while others experienced the loss of virulence genes. Furthermore, it was observed that multidrug-resistant *S*. Newport and Infantis isolates retained a substantial portion of the virulence genes. Some genotypically sensitive *S*. Infantis isolates were also consistently devoid of certain virulence genes, including *fyuA*, the *irp* gene cluster, and the *ybt* gene cluster. Understanding these complicated adaptations in *S. enterica* is crucial for public health efforts, as it highlights the need for science-based AMU and surveillance to track the emergence of AMR.

### Whole-genome phylogeny

The whole-genome maximum-likelihood phylogenetic trees with sequence types, and plasmid, integron, AMR, and virulence patterns of *S*. Typhimurium, Newport, and Infantis isolates from Latin American surface waters are shown in Fig. S1. Figure S1A illustrates the formation of two major well-defined clades (Clades I and II) on the phylogenetic tree comprising 106 *S*. Typhimurium isolates, exhibiting a broad range of SNPs from 0 to 1,552. Clade I consisted of 42 isolates, encompassing 32 Chilean isolates, five Mexican isolates, and five Brazilian isolates, all sharing the same ST (19) (Table S4). The SNPs among isolates in Clade I ranged from 1 to 1,015. The largest SNP difference (1,015) was detected between two Chilean isolates. Clade II comprised 64 isolates, with 30 Chilean isolates, 23 Mexican isolates, and 11 Brazilian isolates, representing a diverse range of STs, including 19, 34, 99, 213, and 2072 (Table S4). The range of SNPs among isolates in Clade II extended from 0 to 1,221. In a manner similar to Clade I, the highest SNP variation in Clade II (1,221) was also identified between two Chilean isolates. Interestingly, 23 *S*. Typhimurium isolates from Chile and Mexico exhibiting potential fitness costs imposed by either ARGs or virulence genes formed a single cluster on the tree. This cluster included three distinct STs, including 19, 34, and 213 (Table S4). The SNP variation among isolates in this cluster spanned from 0 to 443. The evolutionary sacrifice events appeared to have shaped the genetic similarity within the cluster. This clustering further highlights a shared phenomenon where acquiring ARGs has led to the selective loss or reduced presence of specific virulence genes.

The phylogenetic tree with 161 *S*. Newport isolates reveals the presence of two major clades, denoted as Clades I and II (Fig. S1B). These clades exhibit a significant diversity in SNPs, ranging from 0 to 1,167. Clade I included 79 isolates, comprising 15 Chilean isolates, 34 Mexican isolates, and 31 Brazilian isolates, showcasing a wide variety of STs, including 118, 164, and 2370 (Table S4). The SNPs among isolates in Clade I ranged from 0 to 102. The largest SNP difference (102) was detected between one Mexican isolate and one Brazilian isolate. Clade II included 82 isolates, consisting of 36 Chilean isolates, 39 Mexican isolates, and 7 Brazilian isolates, representing a diverse spectrum of STs, such as 31, 45, 132, and 7815 (Table S4). The SNPs among isolates in Clade II ranged from 0 to 956. The largest SNP difference (956) was detected between one Chilean isolate and one Mexican isolate. Multidrug-resistant *S*. Newport isolates formed a single cluster of 30 isolates, including one Chilean isolate and 29 Mexican isolates. All isolates within this cluster shared the same ST (132) (Table S4). Notably, we detected a genetic relatedness between one Chilean isolate (CFSAN125066) and two Mexican isolates (CFSAN115844 and CFSAN121391) in this cluster, with a surprisingly minimal genetic distance of four and 13 SNPs, respectively. Intriguingly, the closely related isolates also exhibited congruent MDR, plasmid (IncR), and virulence patterns. It should be emphasized that the sampling site for CFSAN115844 is not directly connected to the one for CFSAN121391 (11 SNPs). According to the NCBI Pathogen Detection database, these isolates were identified within the SNP cluster PDS000007781.917 and displayed a close relationship with two Mexican isolates originating from beef-based dog food (bully stick) samples (CFSAN125066 and CFSAN115844: two SNPs; CFSAN121391: 11 SNPs).

CFSAN125066 was collected from a sampling site located in the small urban area of Talagante, which is surrounded by agricultural zones and situated within a slum on the riverbed of the Mapocho River. Livestock such as cows and horses are frequently observed drinking water from the Mapocho River in this area. CFSAN115844 was collected from a canal located in the Xochimilco municipality of Mexico City. The canal is not only a tourist attraction but also vital to producing flowers and vegetables for human consumption. Treated wastewater from the city is used to replenish the canal. However, it is possible that the treatment may not be effective enough to prevent water contamination. In addition, the presence of animals, particularly pets and birds, with direct access to these waters could also contribute to potential contamination. CFSAN121391 was obtained from a river in Tlaxcala State, located more than 100 km to the south of Mexico City. This area is in close proximity to extensive agricultural regions. While Tlaxcala primarily emphasizes vegetable production, there could be some small-scale livestock farms in this area. This site is easily accessible to animals for drinking water.

The striking close relatedness of these three isolates from Chile and Mexico, as exemplified by their nearly identical genomes, raises intriguing questions about the potential mechanisms underpinning their genetic similarity. Most importantly, these isolates shared the noteworthy feature of MDR. The presence of isolates from Chile and Mexico within the same SNP cluster, as cataloged in the NCBI Pathogen Detection database, hints at the potential global dissemination of these multidrug-resistant isolates. This observation challenges the traditional understanding of geographical divergence in bacterial populations. Our findings, therefore, emphasize the importance of a comprehensive approach to *S. enterica* surveillance, as factors beyond geographical boundaries, such as international trade, human travels, animal movements, overlapping ecological niches, and potentially shared sources of contamination, may collectively play roles in shaping the genomic landscape of *S. enterica* (79). Ultimately, the genomic relatedness of *S. enterica* isolates from different countries underscores the need for further investigation into these factors influencing the global distribution and genomic relatedness of this pathogen.

In Fig. S1C, we identified two major clades (I and II) on the phylogenetic tree of 112 *S*. Infantis isolates, showcasing a range of SNPs from 0 to 411. Clade I consisted of 24 isolates, encompassing 6 Chilean isolates and 18 Brazilian isolates, all with STs of 32 and 1032 (Table S4). The SNP variation among isolates in Clade I spanned from 1 to 286, with the greatest SNP divergence (286) observed between two Brazilian isolates. Clade II comprised 88 isolates, with 66 from Chile and 22 from Mexico, sharing STs of 32 and 9835. Within Clade II, the range of SNP variation among isolates extended from 0 to 270, with the most notable SNP difference (270) observed between two Mexican isolates. A total of 58 *S*. Infantis isolates with genotypic MDR clustered together, including 52 Chilean and 6 Mexican isolates. Within this cluster, the range of SNPs among isolates varied from 0 to 103. Interestingly, these isolates in the MDR cluster possessed unique virulence genes not found in the other 55 isolates. These genes included *fyuA*, which encodes the pesticin/yersiniabactin receptor protein, the *irp* gene cluster responsible for encoding the yersiniabactin biosynthetic protein, and the *ybt* gene cluster associated with the yersiniabactin siderophore biosynthetic protein.

### cgMLST

**Fig 3:**
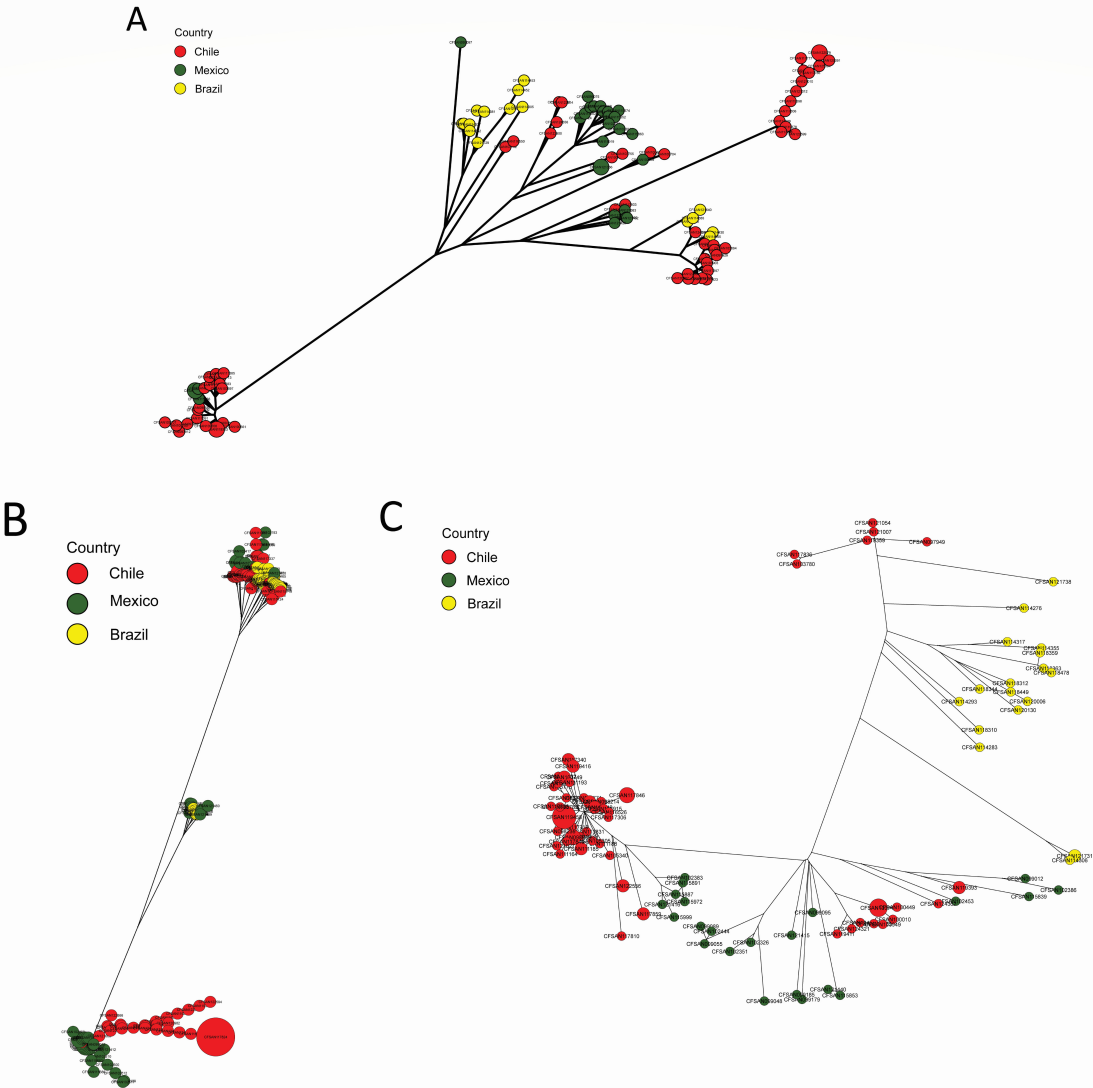
cgMLST-based minimum spanning tree of *Salmonella enterica* serotypes Typhimurium (**A**), Newport (**B**), and Infantis (**C**) isolates from Latin American surface waters. The node area in each tree is proportional to the number of isolates in the area.

Our cgMLST analysis not only reinforced the clustering patterns observed in the whole-genome phylogenetic analysis but also allowed for a more focused examination of serotype-specific genetic diversity (Fig. 3). Specifically, within each of the cgMLST-based minimum-spanning trees in Fig. 3, we observed two major clades for each serotype. This observation suggests that the genetic variations and relatedness of isolates within each serotype were maintained across both analyses, highlighting the consistency and accuracy of our findings.

### Pan-genome

**Fig 4:**
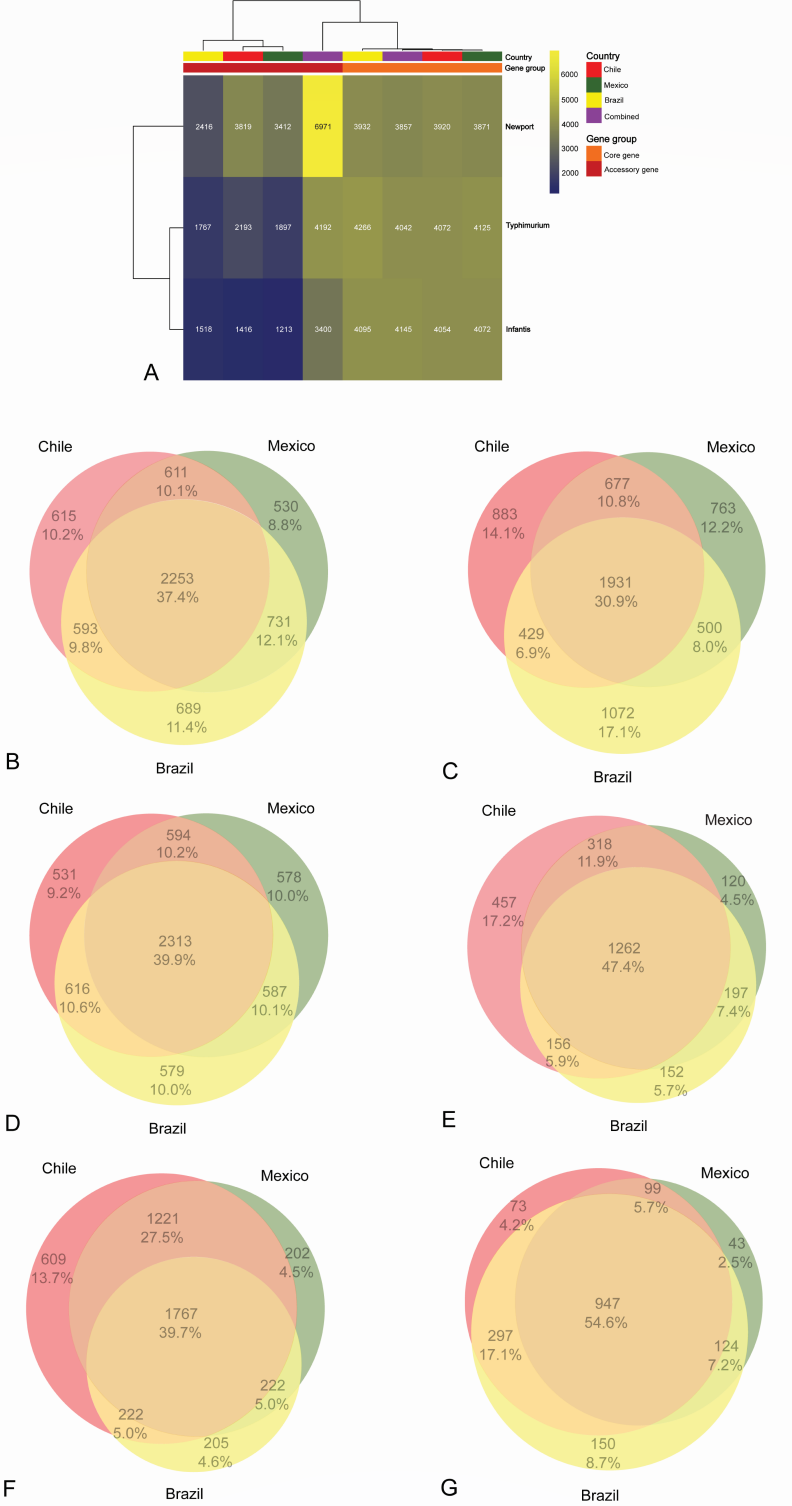
Numbers of core and accessory genes (**A**), and area-proportional Venn diagrams of core (B, C, and D, respectively) and accessory (E, F, and G, respectively) genes of *Salmonella enterica* serotypes Typhimurium, Newport, and Infantis isolates from Latin American surface waters. The intersection area in each diagram is proportional to the percentage of isolates in the area.

Our pan-genome analysis unveiled distinctive genomic profiles among the isolates derived from Chile, Mexico, and Brazil, shedding light on the potential genetic adaptations to local environments. As revealed in Fig. 4A, higher counts of core genes were found in *S*. Typhimurium, Newport, and Infantis isolates from Brazil, with 4,266, 3,932, and 4,095 core genes, respectively. This observation suggests that Brazilian isolates may share a more conserved genomic core. It is important to note that core genes typically encode fundamental cellular functions (80), and their higher presence among these isolates may indicate that these functions are essential for adapting to and surviving in the local conditions prevalent in those regions. The genetic diversity observed among the isolates from different countries led to the identification of distinct sets of core genes for each country. While core genes are generally conserved across isolates, the variations in genomic content among isolates from different geographical regions can result in the identification of unique sets of core genes for each country. This diversity can be influenced by factors such as regional differences in bacterial populations, environmental conditions, and evolutionary processes (81). In summary, different core genes observed in Chile, Mexico, and Brazil reflect the genomic diversity within the isolates collected from these specific geographical locations. The identification of country-specific core genes allows us to explore the unique genomic features of *S. enterica* populations in each region. While our study provides valuable insights into the pan-genome diversity of *S*. Typhimurium, Newport, and Infantis isolates from Latin American surface waters, it is crucial to acknowledge the potential influence of sample size on our findings. Notably, the smaller number of isolates from Brazil (*n* = 71) in comparison to Chile (*n* = 185) and Mexico (*n* = 123) may have contributed to an overestimation of the count of core genes. This limitation underscores the need for a cautious interpretation of these results.

By contrast, *S*. Typhimurium and Newport isolates from Chile and Infantis isolates from Brazil exhibited higher counts of accessory genes (2,193, 3,819, and 1,518, respectively) (Fig. 4A). The elevated number of accessory genes in these isolates suggests that the isolates may have promoted the acquisition and retention of genes that offer the isolates unique traits that facilitate niche-specific adaptations to surface waters and thrive in the local environment. The proportions of overlapped and distinct core and accessory genes between isolates from each pair of the three countries did not show significant differences (*P* > 0.05). Nonetheless, higher proportions of overlapped accessory genes were observed between *S*. Typhimurium isolates from Chile and Mexico (318, 11.9%), *S*. Newport isolates from Chile and Mexico (1,221, 27.5%), and *S*. Infantis isolates from Chile and Brazil (297, 17.1%) (Fig. 4B through G).

**Fig 5:**
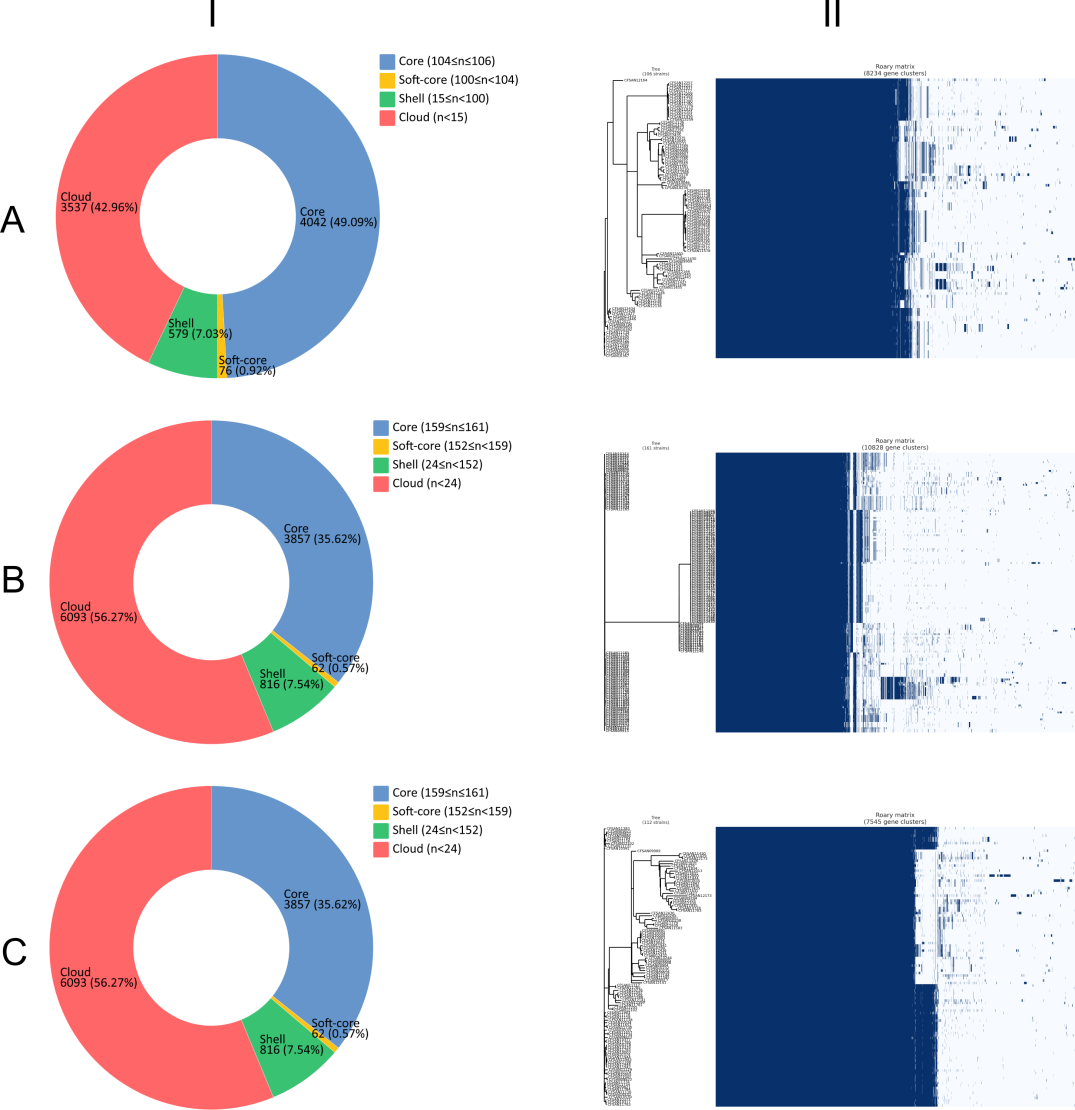
Pan-genome compositions (**I**), and core-genome phylogenetic trees aligned with the matrices of the presence and absence of core and accessory genes (II) of *Salmonella enterica* serotypes Typhimurium (**A**), Newport (**B**), and Infantis (**C**) isolates from Latin American surface waters.

As shown in Fig. 5, the results from our pan-genome analysis involving isolates from the three countries unveil interesting patterns. *S*. Typhimurium and Infantis isolates had a greater number of core genes (4,042 and 4,145, respectively) than *S*. Newport isolates (3,857). Meanwhile, *S*. Typhimurium and Infantis isolates had higher proportions of overlapped core (2,253, 37.4% and 2,313, 39.9%, respectively) and accessory (1,262, 47.4% and 947, 54.6%, respectively) genes among the three countries (Fig. 4B through G). This implies that *S*. Typhimurium and Infantis isolates shared a more stable genomic core across countries, highlighting their conserved genetic elements that likely play pivotal roles in their survival and adaptability. Conversely, *S*. Newport isolates contained more accessory genes (6,971) than *S*. Typhimurium and Infantis isolates (4,192 and 3,400, respectively). In our investigation, the observed larger number of accessory genes in *S*. Newport compared to *S*. Typhimurium and Infantis can be attributed to the presence of three distinct lineages within *S*. Newport (82). The extended evolutionary divergence among these lineages can contribute to a more expansive accessory genome, reflecting the genetic diversity accumulated over their respective evolutionary histories. This distinction aligns with the complexities introduced by the diverse lineages within *S*. Newport, providing a nuanced perspective on the observed genomic differences among *S. enterica* serotypes. Moreover, we acknowledge that the observed abundance of accessory genes in *S*. Newport compared to the other two serotypes, *S*. Typhimurium and Infantis, can also be attributed, in part, to the larger number of *S*. Newport isolates in our study. The influence of sample size on pan-genome analysis is a crucial consideration and the unequal representation of serotypes may introduce variability in accessory gene prevalence.

The outcomes of our pan-genome analysis provide insights into the genomic diversity among *S*. Typhimurium, Newport, and Infantis isolates from Chile, Mexico, and Brazil, offering a glimpse into potential genetic adaptations to their respective environments. The variations in core and accessory gene count among isolates from different countries and serotypes suggest that *S. enterica* has undergone distinct genetic adaptations to their local environments, reflecting the complexity and diversity of surface water ecosystems across Chile, Mexico, and Brazil. These findings warrant further investigation to uncover the specific genetic traits and ecological factors contributing to these observed patterns.

### Conclusions

Our comprehensive genomic analysis of *S*. Typhimurium, Newport, and Infantis from surface waters across Chile, Mexico, and Brazil has unveiled a complex landscape of genomic diversity. Our findings highlight the critical role that environmental reservoirs of *S. enterica* play in public health, reinforcing the importance of continued surveillance and good agricultural practices aimed at minimizing the transmission of this pathogen from surface waters to humans through various pathways. By revealing the genetic makeup of these isolates, we also gain insights into potential risks for MDR dissemination. For future studies, it would be valuable to explore the genetic relatedness and potential transmission routes between surface water and clinical isolates of *S*. Typhimurium, Newport, and Infantis, which can provide insights into the public health implications of environmental reservoirs in the epidemiology of salmonellosis.

## Data Availability

Raw reads were deposited into the Sequence Read Archive (SRA) database hosted by the NCBI under BioProject accession numbers PRJNA186035 and PRJNA560080.

## SUPPLEMENTAL MATERIAL

Figure S1A spectrum.00047-24-s0001.docx Whole-genome maximum-likelihood phylogenetic tree with sequence types, and plasmid, integron, genotypic antimicrobial resistance, and virulence patterns of *Salmonella enterica* serotype Typhimurium isolates.

Figure S1B spectrum.00047-24-s0002.docx Whole-genome maximum-likelihood phylogenetic tree with sequence types, and plasmid, integron, genotypic antimicrobial resistance, and virulence patterns of *Salmonella enterica* serotype Newport isolates.

Figure S1C spectrum.00047-24-s0003.docx Whole-genome maximum-likelihood phylogenetic tree with sequence types, and plasmid, integron, genotypic antimicrobial resistance, and virulence patterns of *Salmonella enterica* serotype Infantis isolates.

Table S1 spectrum.00047-24-s0004.xlsx Metadata of *Salmonella enterica* serotypes Typhimurium, Newport, and Infantis isolates.

Table S2 spectrum.00047-24-s0005.xlsx Plasmid presence and absence of *Salmonella enterica* serotypes Typhimurium, Newport, and Infantis isolates.

Table S3 spectrum.00047-24-s0006.xlsx Integron presence and absence of *Salmonella enterica* serotypes Typhimurium, Newport, and Infantis isolates.

Table S4 spectrum.00047-24-s0007.xlsx Sequence types of *Salmonella enterica* serotypes Typhimurium, Newport, and Infantis isolates.

ASM does not own the copyrights to Supplemental Material that may be linked to, or accessed through, an article. The authors have granted ASM a non-exclusive, world-wide license to publish the Supplemental Material files. Please contact the corresponding author directly for reuse.
