# Supplementary material for: Unveiling the genomic landscape of Salmonella enterica serotypes Typhimurium, Newport, and Infantis in Latin American surface waters: a comparative analysis
Source: Microbiol Spectr. 2024 Mar 28;12(5):e00047-24. doi: 10.1128/spectrum.00047-24 (PMC11064523; doi:10.1128/spectrum.00047-24)
Supplement: Figure S1A — Whole-genome maximum-likelihood phylogenetic tree with sequence types, and plasmid, integron, genotypic antimicrobial resistance, and virulence patterns of Salmonella enterica serotype Typhimurium isolates. [file spectrum.00047-24-s0001.docx]

**Figure S1A** Whole-genome maximum-likelihood phylogenetic tree with sequence types, and plasmid, integron, genotypic antimicrobial resistance (AMR), and virulence patterns of *Salmonella* *enterica* serotype Typhimurium isolates from Latin American surface waters. The scale bar stands for the number of substitutions per site.
